# International Students’ Academic Adjustment in South Korea: A Qualitative Case Study

**DOI:** 10.3390/bs16081390

**Published:** 2026-08-13

**Authors:** Yuanying Jin, Jieun Kang, Xiaocheng Wang

**Affiliations:** 1 Department of Education, College of Humanities, Sejong University, Seoul 05006, Republic of Korea; yyjin@sejong.ac.kr; 2 Department of Education, Korea University, Seoul 02841, Republic of Korea; troykuku1008@naver.com; 3 Department of Education, School of Humanities, Jiangnan University, Wuxi 214122, China

**Keywords:** academic adjustment, qualitative case study, international students

## Abstract

Although research on international students’ academic experiences in South Korean universities has steadily grown, relatively little attention has been paid to how undergraduate international students from diverse national backgrounds experience and navigate academic adjustment in their everyday university lives. To address this gap, this qualitative case study draws on the collective narratives of sixteen undergraduate international students enrolled at South Korean universities. The study examines how participants define academic adjustment, what factors they perceive as influencing their adjustment, and how they navigate the process of becoming academically adjusted. Data analysis generated three overarching themes and nine categories: (1) becoming a competent, connected and self-directed student; (2) negotiating adjustment amid barriers and support; (3) taking ownership through action and reflection. The findings offer implications for policymakers and educators seeking to support undergraduate international students’ academic adjustment in South Korean higher education.

## 1. Introduction

Over the past two decades, South Korean universities have experienced a substantial change in student composition. Although domestic students remain the dominant group within overall university enrollment, the rapid growth in international student enrollment has made campuses increasingly internationalized. In 2025, the number of degree-seeking international undergraduate students reached 120,150, representing a 12.2-fold increase from 2005. Their share of total undergraduate enrollment also rose markedly, from 0.3% in 2005 to 4.5% in 2025 (Korean Educational Statistics Service, 2025). The largest source countries were China, Vietnam, Nepal, Uzbekistan, and Mongolia, which accounted for 31.6%, 27.2%, 9.2%, 9.1%, and 5.8% of undergraduate international students, respectively (Korean Educational Development Institute, 2025).

The growth of international student enrollment in South Korea reflects both national policy initiatives and institutional adaptation. Since 2004, the Korean government has pursued a series of recruitment policies to promote the internationalization of higher education, beginning with the Study Korea Project, which targeted 50,000 international students by 2010. This was followed by the Study Korea 2020 Project in 2012, which set a goal of 200,000 students by 2020, and the Study Korea 300K Project in 2023, which aims to attract 300,000 international students by 2027 and position Korea among the world’s top 10 study destinations (Y. Kim, 2024). Alongside these expansion efforts, the government introduced the International Education Quality Assurance System (IEQAS) in 2012 to strengthen oversight of the quality of universities’ international education practices (Y. J. Oh & Park, 2018). At the same time, universities have expanded international admissions in response to the shrinking college-age population and the resulting financial strain. Reflecting this demographic trend, South Korea’s total fertility rate declined from 1.48 in 2000 to 0.8 in 2025 (Ministry of Data and Statistics, 2025).

Although studying overseas can provide important academic, linguistic, and personal opportunities, it also entails substantial financial, academic, and emotional challenges. Previous studies have reported that international students in South Korea experience a range of psychological difficulties, including discrimination, acculturative stress, interpersonal difficulties, and financial burden (Y. Lee et al., 2022; H. Y. Oh & Lee, 2018). In addition, they face various academic challenges, such as language barriers, poor academic performance, limited academic engagement, difficulty adjusting to a new educational system, challenges in participating in team projects, and limited interaction with local students (J. Kim & Sim, 2018; Y. Lee et al., 2022; Mun, 2018; Vanchinkhuu & Shin, 2023). These difficulties can contribute to academic probation (Sung et al., 2018) and, in some cases, dropout (I. C. Shin et al., 2018). For instance, in 2025, the dropout rate of degree-seeking undergraduate international students at a private four-year university in Seoul was 15.3%, approximately three times that of local students at the same institution (Korean University Information System, 2025b). Since academic adjustment is a key aspect of international students’ overall adjustment (Mori, 2000; Poyrazli & Grahame, 2007), their academic adjustment experiences deserve greater scholarly attention.

Against this backdrop, the present study examines the academic adjustment of undergraduate international students in South Korea. Adjustment refers to the process through which individuals actively interact with their environment, either by changing themselves or by modifying the environment to meet its demands (Lazarus, 1976). Drawing on Baker and Siryk (1984), academic adjustment can be understood as the extent to which students successfully respond to the educational demands of the university environment. This is reflected in their attitudes toward academic goals and tasks, their level of effort, the effectiveness of their academic functioning, and their satisfaction with the academic environment. Gu et al. (2010) further suggest that international students’ academic adjustment emerges from the interplay of language proficiency, social interaction, personal development, and academic outcomes. Drawing on these perspectives, the present study approached academic adjustment as a multidimensional process that extends beyond academic achievement to include students’ attitudes, motivation, social and psychological experiences, and interactions with the educational environment.

Prior research on the academic experiences of international students in the South Korean context can be broadly grouped into several categories. A considerable body of research has examined factors influencing academic outcomes such as academic achievement, academic adjustment, and academic satisfaction. J. Noh (2020), for example, identified major factors affecting international students’ academic achievement through a literature review, expert consultation, a survey, and factor analysis. The study found that academic language, cognitive factors, problem-solving ability, self-efficacy, anxiety, social support, and interpersonal relationships were among the key determinants of academic achievement. Building on this line of inquiry, other studies have examined a broad range of individual and contextual variables related to academic outcomes. For example, some studies have explored the effects of academic self-efficacy, academic emotion regulation, and academic motivation on academic achievement (Chen & Kang, 2018; Y. Lee, 2019), whereas others have investigated the effects of Korean language proficiency, health status, awareness of Korean popular culture, acculturative stress, and psychological well-being on academic adjustment (Ha & Zhu, 2016; Jang, 2018; Li & Ahn, 2016; Vanchinkhuu & Shin, 2023). Still others have considered the roles of life stress, acculturative stress, and instructors’ transformational leadership in shaping academic satisfaction (X. Jin & Hahm, 2018; Lim & Lee, 2017).

Some studies have focused on the current status of international students’ academic adjustment. For example, S. Lee and Na (2018) investigated this issue through interviews with faculty members, international students, and domestic students, along with a survey of international students, to inform the development of general education courses for this population. Their findings showed that international students experienced academic difficulties, with Korean language proficiency and insufficient basic academic skills identified as major barriers to academic adjustment. Similarly, Bak (2024) examined the difficulties and needs of international students during academic adjustment, finding that they experienced language barriers and acculturative stress and that language education was their most pressing need. I. C. Shin et al. (2018), meanwhile, investigated international student dropout rates and their contributing factors, identifying university prestige, dormitory availability, and language proficiency as significant predictors.

Another strand of research has examined the development and implementation of interventions for international students, including study skills training, stress management, study coaching, and tutoring programs, as well as their effectiveness (Ahn et al., 2023; H. J. Hong et al., 2013; H. J. Kim & Cho, 2018; Yoon & Shim, 2019). Furthermore, some studies have focused on the development of academic assessment tools. E. Park and Park (2017), for example, developed a diagnostic instrument for academic life adjustment among international students, while H. Jin and Chi (2017) developed and validated a scale measuring dropout intention among Chinese international students.

Existing research has also examined overall scholarly trends in this area (Y. Y. Jin et al., 2021; D. H. Shin & Kim, 2020). For example, D. H. Shin and Kim (2020) used text mining and topic modeling to examine research trends on international students, concluding that the literature has largely approached them from two perspectives: as learners and as immigrants. This suggests that research on this group has considered not only their recruitment and academic support but also broader issues of migration and adjustment. Y. Y. Jin et al. (2021) analyzed 67 studies published in South Korea between 2011 and 2020 and reported that nearly half were conducted by researchers in language and cultural studies, whereas only about 28% were carried out by researchers in education. This suggests that both research and support practices have tended to conceptualize international students’ difficulties primarily in linguistic terms, with a corresponding emphasis on improving Korean language proficiency.

Qualitative studies on academic adjustment have also been conducted. However, these studies have largely focused on students from specific countries or regions, such as Southeast Asia (J. Kim, 2013) or Uzbekistan (Galanova, 2018); on extreme cases of academic adjustment such as academic probation (Hwang et al., 2016; M. J. Lee et al., 2019; Sung et al., 2018); or on the academic adjustment of graduate students (Y. K. Lee, 2014).

Although research on international students’ academic experiences has grown steadily, it has largely concentrated on factors related to academic outcomes, the challenges of adjustment, and the experiences of specific groups or exceptional cases. In contrast, relatively little attention has been paid to how undergraduate international students from diverse national backgrounds actually experience and navigate academic adjustment in their everyday university lives. This gap is important because academic adjustment is embedded in the specific context of university life in Korea, and the undergraduate experience differs from the graduate experience in important environmental and developmental respects. Moreover, academic probation represents a special and extreme condition rather than a typical form of academic adjustment, which limits its usefulness for understanding the broader experiences of undergraduate international students.

Accordingly, there is a need for an in-depth investigation of how undergraduate international students from diverse national backgrounds adjust academically within the context of university life in Korea, including the difficulties they encounter and the meanings they make of those experiences. Such research would contribute to a more grounded and nuanced understanding of international students’ academic adjustment and provide an important foundation for developing educational support strategies tailored to the characteristics and needs of undergraduate international students.

This study adopted a qualitative case study approach to explore the academic adjustment experiences of undergraduate international students at Korean universities. Specifically, it sought to examine how these students perceive their academic adjustment, the factors that influence it, and the process through which it unfolds. The study was guided by the following research questions:

First, how do undergraduate international students in South Korea perceive their academic adjustment at Korean universities?

Second, how do undergraduate international students in South Korea perceive the factors influencing their academic adjustment?

Third, how do undergraduate international students in South Korea perceive the process of academic adjustment at Korean universities?

## 2. Methods

### 2.1. Research Approach

This study adopted an exploratory and inductive qualitative approach. Given this orientation, the analysis was not guided by a fixed theoretical framework but was intended to develop insights from participants’ accounts rather than test a predetermined theory (e.g., Patton, 1990). Within this exploratory orientation, a qualitative case study design was employed to explore the academic adjustment experiences of international students in South Korean universities, as this phenomenon is embedded within institutional and sociocultural contexts, and the boundaries between the phenomenon and its context are not clearly evident (Yin, 2009). Drawing on Yin’s (2009) conceptualization of case boundaries and embedded units of analysis—rather than his theory-testing orientation—this study adopted an embedded single-case design. The bounded case was the experience of international students adjusting to academic life in South Korean higher education institutions, with individual participants serving as embedded units of analysis. The analysis focused on identifying shared patterns of meaning among the participants in this case.

### 2.2. Recruitment

Purposive sampling involves the deliberate selection of information-rich cases that can provide meaningful insight into the research questions (Patton, 1990). Accordingly, we recruited international undergraduate students with direct experience of academic adjustment in South Korean universities. Two inclusion criteria were applied: (a) participants had completed at least one semester of undergraduate study to ensure adequate exposure to the university academic environment, and (b) they were able to communicate their experiences in Korean, English, Chinese or Vietnamese. These language options reflected both the linguistic context of South Korean universities and the composition of the international student population. In qualitative research, conducting interviews in participants’ preferred language is important because it enables them to describe their experiences more comfortably and in greater depth (Temple & Young, 2004). Eligibility was assessed through a brief pre-interview (approximately 10 min) conducted with each prospective participant prior to the formal interview. This screening ensured that participants met the inclusion criteria and were able to articulate their academic experiences in sufficient detail.

The study received approval from the Institutional Review Board (IRB) of the first author’s affiliated university prior to recruitment. Sixteen international undergraduate students from five countries and six universities participated in the study. Three universities were in Seoul, one in a satellite city near Seoul and two in other regions of South Korea. All participating institutions were private, research-intensive universities with relatively high proportions of international students. Participants were recruited primarily through international student communities, with additional participants identified via snowball sampling. Among the 16 participants, seven were men. All were enrolled in Korean-mediated courses, and their ages ranged from 21 to 26 years. Participants represented diverse majors and levels of Korean language proficiency (TOPIK Levels 1–6), as well as varying lengths of residence in South Korea (1–6 years), allowing for an examination of heterogeneous patterns of adjustment. Demographic characteristics are presented in Table 1. Written informed consent was obtained from all participants.

### 2.3. Data Collection

Prior to the interviews, participants completed a brief questionnaire collecting demographic and academic information, including nationality, gender, GPA, major, length of stay in South Korea, and Korean language proficiency. At this stage, the researcher also provided participants with the interview protocol (see Table 2) to ensure that they were familiar with the scope and focus of the interview. Semi-structured interviews were conducted using a protocol developed based on previous research on international students’ academic adjustment and refined based on feedback from four experts in education. The interview protocol was structured around three interrelated domains: (a) participants’ perceptions and definitions of successful academic adjustment, (b) factors perceived as facilitating or hindering their adjustment process, and (c) their lived experiences of performing academic tasks and interacting within the university context. This structure ensured close alignment between the research aims and the data collection procedures. Interviews were conducted online and lasted between 30 and 95 min (*M* = 50 min). All interviews were audio-recorded with participants’ consent, transcribed verbatim, and de-identified to protect confidentiality. The de-identified transcripts and research memos were imported into MAXQDA 2020 for systematic data management.

### 2.4. Data Analysis

Data were analyzed using Braun and Clarke’s (2006) reflexive thematic analysis with an inductive, latent orientation. Themes were generated inductively from participants’ narratives, rather than being shaped by predetermined analytic categories. At the same time, analysis was informed by existing theoretical perspectives on academic adjustment and acculturation to ensure conceptual sensitivity. The analytic process followed six recursive phases (Braun & Clarke, 2006). First, the researcher engaged in repeated reading of the transcripts to achieve familiarity with the dataset and generated initial analytic memos. Second, initial codes were generated across the entire dataset, capturing meaningful segments related to adjustment experiences, coping strategies, and contextual influences. Third, codes were grouped into candidate themes based on conceptual similarity. Fourth, themes were reviewed and refined by examining their coherence within individual cases and across cases. During this stage, particular attention was paid to identifying both shared structural patterns and meaningful variations among participants. Fifth, themes were defined and named to ensure conceptual clarity and distinctiveness. Finally, representative excerpts were selected to illustrate each theme in the results. Throughout the analysis, coding decisions, theme development, and interpretive reflections were documented to maintain analytic transparency.

### 2.5. Trustworthiness

To enhance the credibility of the findings, participants were invited to review interpretive summaries and provide feedback on emerging themes. Their feedback was considered in refining analytic interpretations and clarifying potential misunderstandings. In addition, peer debriefing was conducted throughout the analytic process to critically examine developing interpretations and to minimize potential bias. An audit trail was systematically maintained to document coding decisions, theme development, analytic memos, and revisions, thereby strengthening transparency and dependability.

### 2.6. Researcher Reflexivity

This study was conducted within a constructivist–interpretivist paradigm (Ponterotto, 2005), which acknowledges that researchers’ backgrounds and experiences inevitably shape the research process. All authors have prior academic or professional experience related to international students in South Korea. The first author has training in educational counselling and experience providing psychological support to international students; the co-author, trained in counselling education, has been involved in research and practice with international student populations, and the corresponding author has conducted sustained research involving international students. Given these professional engagements, we remained attentive to how our prior knowledge and assumptions might influence data interpretation. Throughout data collection and analysis, we engaged in ongoing reflexive discussion and memo-writing to critically examine our interpretive positions. This process enhanced analytic transparency and contributed to the credibility of the findings.

## 3. Results and Discussion

**Figure 1:**
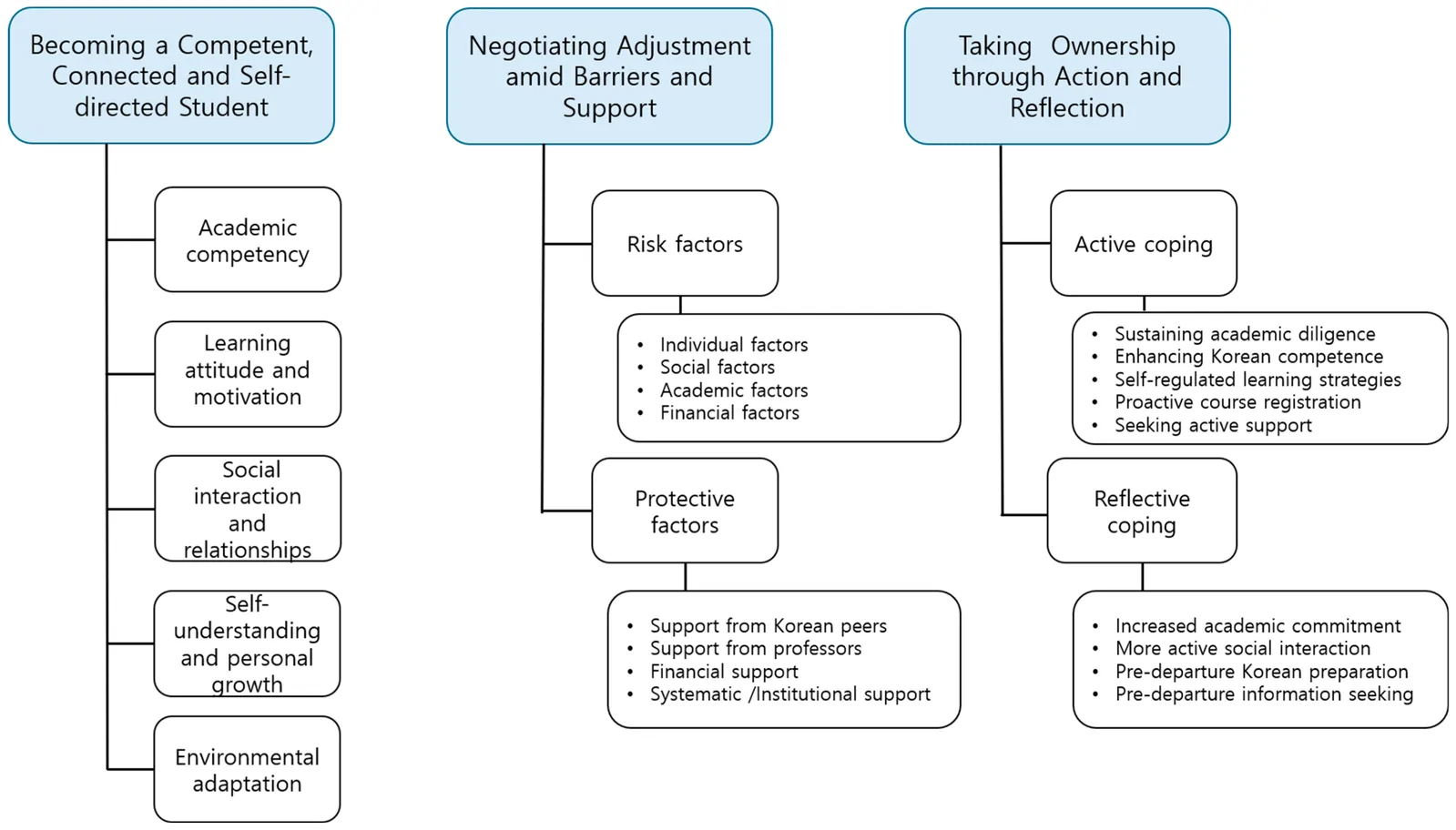
Summary of themes and categories.

Three overarching themes and nine categories emerged from the data analysis: (1) becoming a competent, connected and self-directed student; (2) negotiating adjustment amid barriers and support; (3) taking ownership through action and reflection (see Figure 1 and Appendix A).

### 3.1. Theme 1: Becoming a Competent, Connected and Self-Directed Student

The first theme captures how participants understood what it meant to adjust successfully to academic life in South Korea. Five categories emerged from the data analysis as key indicators of successful academic adjustment.

#### 3.1.1. Academic Competency

Participants consistently highlighted academic competence as a central indicator of academic adjustment. They described successful academic adjustment as the ability to understand and keep pace with coursework. This included comprehending instructors’ lectures, becoming accustomed to instructional and learning styles, and staying informed about important classroom announcements and notices. Participants also viewed Korean language proficiency and objective academic outcomes as key indicators of academic adjustment. Additionally, objective academic achievement was considered an important indicator of academic competence. This included improved grades, strong performance and acquiring new knowledge. As one participant explained, ‘Ultimately, getting good grades is what matters most to me’ (Participant 10).

#### 3.1.2. Learning Attitude and Motivation

Participants also emphasized learning attitude and motivation as important components of academic adjustment. They highlighted diligence, including conscientious engagement with coursework, avoidance of procrastination, active participation in team projects, and the investment of additional effort in assignments. They further highlighted the value of previewing and reviewing course content to enhance comprehension. Enjoyment of learning was likewise identified as a key indicator: participants described valuing engagement that stems from genuine interest, taking pleasure in the learning process, and approaching their studies with a sense of ease and composure. Maintaining self-efficacy was also identified as a central aspect of adjustment. Participants referenced perceiving themselves as capable of completing assignments independently, managing negative emotions when learning became challenging, expressing their ideas confidently in class and during team projects, and sustaining high levels of learning motivation. As participant 15 noted:

I think one of the most important things in adjusting well is to speak up often and not be afraid of giving presentations or talking in front of others. For example, when a professor asks a question in class, I am usually prepared and have already organized my thoughts, but when I actually try to speak, I get nervous and end up saying nothing. There were many times when I stayed silent for that reason. I think what really matters is having the courage to speak up. Rather than being intimidated by your language ability or thinking that, as an international student, you are somehow behind Korean students, I think it is important to speak confidently.

(P15)

#### 3.1.3. Social Interaction and Relationships

Participants framed academic adjustment as a multidimensional process encompassing not only academic factors but also social adaptation. They considered social interaction and relationships to be important in supporting academic adjustment. In particular, participants emphasized establishing positive relationships with Korean and international peers and maintaining constructive relationships with professors, which helped them feel more at ease academically. Participants also emphasised the importance of maintaining autonomy in social relationships. They noted that forming close relationships with Korean peers could be challenging and that relying too heavily on peers from the same cultural background could be limiting. They therefore sought to take a balanced and independent approach to their peers. Some participants described prioritizing independence by limiting time spent on social interactions they perceived as inefficient. As participant 12 described:

What didn’t really help was spending a lot of time socializing with Korean friends. I don’t think that was necessary. If you spend too much time on social activities that aren’t meaningful, they don’t really help with your studies. They just end up being a waste of time.

(P12)

#### 3.1.4. Self-Acceptance and Self-Directed Personal Growth

The participants understood academic adjustment to be a process of personal development. Rather than comparing themselves with others, particularly their Korean peers, they learned to accept their circumstances and set themselves realistic goals. They also valued embracing challenges and maintaining a positive, proactive mindset when facing difficulties. They believed that this approach had made them more confident, outgoing and self-reliant. As participant 3 explained, ‘Studying abroad has made me think more deeply, and I feel that it has taught me how to take care of myself and make my own decisions’.

#### 3.1.5. Environmental Adaptation

Participants further identified environmental adaptation as an important dimension of academic adjustment. They explained that this involved adapting to the academic environment by becoming familiar with the learning culture of Korean universities and actively participating in campus activities to develop a sense of belonging. It also involved adapting to the broader living environment by balancing part-time work or on-campus activities with academic demands. As participant 15 stated:

I think successful academic adjustment means not just focusing on studying every day, but also getting involved in things like club activities and gaining different life experiences once you’ve taken care of your studies.

(P15)

These findings suggest that academic adjustment should not be understood solely from a cognitive or functional perspective. Rather, it also involves motivational, emotional, social, and environmental dimensions. This finding is consistent with Baker and Siryk’s (1984) work and Gu et al.’s (2010) argument that academic adjustment is multidimensional. It reflects the developmental characteristics of undergraduate students, who are typically in late adolescence or emerging adulthood and are engaged in self-exploration and identity formation. Accordingly, undergraduate international students appear to perceive academic adjustment not as an isolated academic process, but as closely connected to their social, emotional, and environmental adaptation.

These results may be attributed to differences in the purposes of studying abroad between graduate and undergraduate international students. Graduate international students often arrive in Korea with relatively clear academic goals, and their academic adjustment typically involves adapting to unfamiliar teaching and learning styles, acquiring new study skills, improving Korean language proficiency, and developing relationships with peers and professors (D. Kim et al., 2007). In contrast, undergraduate international students tend to view studying abroad in broader terms. In addition to academic achievement, they often seek diverse experiences and interactions with local students to broaden their worldview (Anderson & Lawton, 2015). Thus, for undergraduate international students, academic adjustment is closely intertwined with personal growth, intercultural learning, and social integration.

These findings highlight the need for policymakers and educators to develop and implement integrated support systems that combine academic advising, Korean language support, psychological counseling, peer mentoring, faculty interaction, career guidance, and practical assistance for academic adjustment. For example, rather than maintaining the current fragmented system, in which administrative support is provided by the international office, academic support by individual departments, and language support by the language institute, it is necessary to establish a more coordinated support system for international students that can provide comprehensive assistance in administration, academics, mental health, career development, and cultural adjustment.

### 3.2. Theme 2: Negotiating Adjustment Amid Barriers and Support

Two overarching categories emerged from the analysis under the theme of how participants negotiated their adjustment amid barriers and support: risk factors and protective factors.

#### 3.2.1. Risk Factors

Through interviews, participants described how individual, social, academic, and financial factors hinder their ability to adapt to university learning demands. At the individual level, participants identified low academic self-efficacy, uncertainty about their major, and health-related issues as barriers that limited persistence and confidence in managing academic demands. For example, participant 16 noted:

I felt like I was giving it my 100%, but I was only getting maybe 30 or 40% back, and that was really hard. Mentally, I kept thinking, ‘Why can’t I keep up? Everyone else is doing so well. They’re all so capable, so why am I the only one falling behind?’ That really took a toll on me.

(P16)

In addition, social-level barriers were described as undermining academic engagement. Participants reported acculturative stress and challenges in socializing with local students, which intensified feelings of isolation, and they also noted that passive compatriot peer networks could discourage active academic engagement. As participant 2 described:

Actually, even before that, I had asked a few Korean friends some questions, but most of them seemed like they didn’t really want to interact with foreigners. Whenever I asked something, they would just say things like, ‘The professor already explained that in class,’ or ‘You should listen to the lecture again.’ Of course, I knew the professor had talked about it during class, but I was asking because I still didn’t understand it, so hearing those kinds of responses was a little hurtful.

(P2)

Moreover, academic-level challenges noted by participants included perceptions of less supportive professors, persistent language barriers, and difficulties adapting to the Korean education system. As participant 14 stated:

When I was a freshman, I was preparing for a group presentation in one of my major classes. We had to make a PowerPoint and present it, so I met up with my Korean teammates at a café to discuss it. But at the time, my Korean wasn’t very strong, so I could more or less understand what they were saying, but I had a hard time expressing exactly what I wanted to say.

(P14)

Finally, financial burden was described as a compounding risk factor. Participants emphasized that financial strain caused by difficulty applying for scholarships could restrict the time and energy available for study and undermine academic focus during the adjustment process. For example, participant 3 stated:

At first, my family supported me financially, but later on, I didn’t want to rely on them anymore, so I tried to manage everything on my own. As a result, I ended up spending all my time working part-time jobs just to cover my living expenses, and I didn’t really have the chance to fully experience life in Korea.

(P3)

These findings suggest that international students’ academic adjustment is shaped by the interplay between personal and environmental factors. This is consistent with previous research identifying social, academic, and financial factors as major challenges for international students, including acculturative stress (Li & Ahn, 2016), negative peer influences from co-national peers (D. H. Shin & Kim, 2019), limited professor support (H. Y. Oh & Lee, 2018), language barriers (S. Hong, 2019), and financial burden (Jeong, 2018). Similarly, previous research has identified individual factors such as academic self-efficacy (J. Noh, 2020), career uncertainty (J. A. Park, 2020), and physical health (Jang, 2018) as potential risk factors for adjustment. In the present study, however, most obstacles perceived by participants were located beyond the individual level, a pattern that also aligns with the broader literature. This may indicate that, for undergraduate international students, external challenges that extend beyond individual academic effort are especially salient.

One possible explanation for this pattern lies in the characteristics of the participants. Among the 16 participants, nine had advanced Korean proficiency, corresponding to TOPIK levels 5 or 6. Given that previous studies have identified language proficiency as an important predictor of academic success (Yan & Cui, 2016), these participants may have already reached a relatively high level of adjustment in Korea. As a result, they may have perceived fewer individual-level risk factors and placed greater emphasis on external or contextual challenges.

Another possible explanation is that, except for studies focusing on more extreme cases, such as students on academic probation (Sung et al., 2018), previous research has rarely examined international students’ own voices in depth in the South Korean higher education context. Much of the existing literature has instead approached academic adjustment from the perspective of institutional providers, such as teaching and learning centers in higher education (Ahn et al., 2023). Consequently, commonly discussed factors tend to be external and institutionally visible, such as language difficulties, whereas individual factors, including academic motivation, learning skills, and psychological well-being, have received comparatively less attention. These findings suggest that effective support for international students should take both individual and contextual factors into account. International students’ voices remain underrepresented in the literature, particularly with regard to individual-level factors. Therefore, further research is needed to examine their experiences and needs from their own perspectives.

#### 3.2.2. Protective Factors

Alongside these risks, participants described protective factors that supported successful academic adjustment. Social support from Korean peers and professors was perceived as particularly important, providing both emotional encouragement and instrumental guidance for navigating coursework and campus resources. As participants 10 and 15 noted:

Another important factor is the Korean friends around them. If those friends are welcoming toward international students and actually want to engage with them, the students can feel that they’re cared for and accepted there. As I mentioned earlier, international students often feel disconnected from the society around them, so when someone takes an interest in them and reaches out, it really does make a difference in how satisfied they feel with their academic life. That kind of experience is really important.

(P10)

In addition, support, care and attention from professors, as well as a sense of being treated equally, contributed to the satisfaction of international students with their university experience. As participant 15 explained:

The professors really care about us and give us a lot of help, even after class. For example, if there’s something we don’t understand well, they’ll explain it again after class or go over it with us one more time. They really show a lot of care and attention. In that sense, I’m really grateful that they treat us equally, without any discrimination.

(P15)

Participants also highlighted financial support (e.g., scholarships or other assistance) as a key buffer against stress, allowing greater focus on academic responsibilities. For example, participant 4 stated:

One reason my satisfaction with studying abroad increased was that it was easy for me to find a part-time job, which meant I could pay for my living expenses myself. I liked the fact that I was able to support myself without financial help from my family.

(P4)

Systematic and institutional support were also discussed as stabilizing factors. For example, national health insurance coverage was perceived as reducing uncertainty and enabling participants to manage health concerns without overwhelming financial risk. In addition, satisfaction with campus facilities and a supportive instrumental environment (e.g., spaces and resources conducive to study) were described as facilitating more consistent academic routines. For example, participant 11 noted:

The school facilities are very modern, and resources like the library and e-books have been really helpful for studying. Also, professors in our department publish their own books and use them as learning materials, which is very convenient for us. In addition, there’s a lounge specifically for international students, and they provide personal items like sanitary pads for women, so if someone needs them urgently, they can get them right away. I think that’s a very thoughtful service and a great example of how well the school cares for students.

(P11)

Participants also detailed a range of institutional programs that they believed could meaningfully affect academic adjustment. Courses designed specifically for international students were generally viewed as helpful; however, participants emphasized the need to improve course quality and instructional design to better align with students’ academic needs. Participants further noted that international-student-friendly systems, including administrative services offered in students’ native languages and dedicated support channels for international students, facilitated their adjustment to the university. Finally, academic mentoring programs, Korean language support, career development services, cultural experience programs, psychological support services, and global buddy programs were perceived as potentially helpful; however, participants emphasized that their effectiveness depends on careful implementation to ensure accessibility, appropriate matching, and sustained engagement. As participant 10 described:

Our school has a separate system in place for international students. There is an administrative office specifically for international students, and when we need help with administrative issues or counseling, they are able to provide support by phone in Chinese. In that sense, I think our school is actually fairly good.

(P10)

These results indicate that undergraduate international students’ academic adjustment depends not only on interpersonal resources, such as support from local students and professors, but also on systematic institutional support. This finding confirmed previous studies suggesting that institutional support programs, including counseling services and learning support programs, can facilitate undergraduate students’ adjustment to university life (An & Jang, 2024; H. J. Hong et al., 2013). In addition, the need to provide career development programs for international students has been emphasized in the literature (E. Noh & Lee, 2024; Yoon, 2024). However, the implementation and effectiveness of such programs have rarely been examined in the existing literature. This may be partly because career support services for international students are less prevalent in Korean universities than counselling and academic support services.

Although previous research has highlighted the importance of support from local students and professors, programs that meaningfully involve these actors remain limited in practice. In South Korean higher education, initiatives such as Global Buddy Programs, which aim to promote mutual support between local and international students, and faculty advisor counseling systems have been widely implemented (S. Park & Choi, 2020; Ha & Zhu, 2016). Nevertheless, these initiatives have often been criticized as formalistic rather than substantive, which may partly explain why their effects have been reported inconsistently across studies (Ha & Zhu, 2016).

Recent studies have begun to explore ways to foster more meaningful interactions among international students, local students, and professors. For example, S. Y. Lee et al. (2022) developed an interaction program based on shared interests and personality information between local and international students and found that it was effective in promoting social interaction. Similarly, Y. S. Kang and Lee (2016) conducted a phenomenological qualitative study to examine factors affecting interactions between professors and international students, with particular attention to both groups’ perspectives on multicultural competencies. However, this topic has not yet received sufficient attention in the literature. Future research should therefore further examine how structured interactions among international students, local students, and professors can be designed and implemented more effectively. This line of inquiry is particularly important because such interactions may benefit not only international students but also domestic students by enhancing opportunities for intercultural communication and learning (Parsons, 2010; Williams & Johnson, 2011).

### 3.3. Theme 3: Taking Ownership Through Action and Reflection

Data analysis revealed two main categories that captured how participants took ownership of their adjustment process through action and reflection: active coping and reflective coping.

#### 3.3.1. Active Coping

Participants described using a range of proactive coping strategies to manage academic demands. In particular, they emphasized maintaining academic diligence through the development of consistent study habits and daily routines, the investment of substantial time and effort in their studies, and deliberate efforts to sustain concentration during class. For example, participant 13 stated:

Since the professor uploads the slides the day before class, I try to prepare in advance. I study the textbook, translate any words I don’t know from the PPT, and add notes directly on the slides. During class, if there’s anything I don’t understand, I mark it with a red pen. Then after class, I try to figure it out by searching online or asking the professor. That’s basically how I deal with academic difficulties.

(P13)

Participants also described drawing on a range of resources to strengthen their Korean language proficiency. They sought support from Korean peers, engaged with Korean media for language learning, and made deliberate efforts to learn about and experience Korean culture. In doing so, they continually developed their Korean proficiency, particularly their command of discipline-specific Korean. As participant 13 noted:

I started by watching a lot of Korean news. I watch Korean news every day at 8 a.m., and sometimes I also watch Korean webtoons or variety shows. I pay attention to how they phrase things, and if I hear useful or really good expressions, I write them down and go back to them sometimes. And honestly, one of the most important things I try to do is talk with Korean people more.

(P13)

Participants further described engaging in a range of self-regulated learning strategies to manage academic demands. They explored learning methods that were well suited to their individual needs, set academic goals while sustaining engagement in their studies, sought information independently to deepen their understanding of course content, and attempted to solve academic problems on their own. In addition, they employed time-management strategies to cope with time-related pressures and engaged in self-directed efforts to address areas of academic weakness. For example, participant 11 explained:

I think it can be thought of in two stages. The first stage was helping myself. When I sometimes ran into difficulties, the first thing I asked myself was whether the problem was with the way I was doing things or with my own understanding. If I could work that out on my own, then I did not really need anyone else’s help.

(P11)

Participants also described making strategic course selections to manage academic demands more effectively. They chose courses that aligned with their level of preparedness and enrolled in classes taught in languages with which they were familiar. As participant 1 explained, “The courses I enrolled in were ones I had chosen myself, so I felt that the knowledge I learned in those classes was truly necessary and beneficial for me”.

In addition, participants drew on support from various interpersonal resources, including professors, peers, Korean friends and mentors, senior international students, and, in some cases, private tutors, to navigate difficulties arising in the course of academic adjustment. As participant 2 remarked:

I messaged a Korean friend and asked, ‘I didn’t really understand this assignment—could you explain it a bit more?’ And they were so kind—they stayed up until 2 a.m. and walked me through everything step by step. That class was online, and the professor was speaking so fast that I just couldn’t keep up. My friend summarized the lecture for me and explained exactly what I should focus on. That experience really stuck with me.

(P2)

#### 3.3.2. Reflective Coping

When reflecting on their past experiences, participants indicated that they would have committed themselves more fully to their studies. For example, participant 3 explained, “I would like to speak up more actively in class and spend more time preparing beforehand by reading the materials in advance, studying, and seeking out related resources on my own”. Specifically, they noted that they would have pursued relevant research experiences in professors’ laboratories, devoted greater effort to developing their English proficiency, and maintained a more proactive approach to their academic work.

Participants also reflected that, if they had another opportunity to begin their academic journey, they would engage more proactively with peers, faculty members, and senior compatriots as a way of facilitating their adjustment. As participant 3 reported:

I would probably approach them naturally, first by saying hello or asking about an assignment. In the past, I felt that expanding my social relationships was difficult, and I thought Korean students probably would not really want to be friends with me anyway. But now, I feel that I need to be the one to reach out first. It may be that they also wanted to be friends with me, but did not have the courage to speak first.

(P3)

Participants also stressed that they would have invested more effort in developing their Korean language proficiency before entering their programs, as they perceived it to be a key resource for navigating both academic demands and everyday life. For example, participant 8 stated:

I think I would prepare my language skills better. Being able to communicate with others is one of the most basic and essential skills for living in a foreign country. So I would work more on my language skills and try to practice more with my friends and with local people here.

(P8)

Finally, participants noted that they would have prepared more thoroughly prior to the start of their academic journey. Specifically, they stated that they would have explored their major in greater depth, established a more systematic study plan, and gathered more extensive information about campus life in advance. As participant 4 stated:

And if I could start over, I think I’d choose a different major than the one I’m in now. I’m studying communication, and it focuses a lot on advertising and broadcasting, but I’m more interested in things like event planning. Looking back, I don’t think I researched the major properly before choosing it. I had a TOPIK level 5, so I got a full scholarship for my first semester and was able to enroll right away. But after four semesters, I’ve realized this major doesn’t really match the career path I want.

(P4)

These results suggest that, for undergraduate international students in South Korea, academic adjustment is not a passive process of waiting for support. Rather, it is an active process in which students exercise agency, using self-regulated strategies to cope with the challenges they encounter. However, much of the existing literature in the South Korean context has focused on addressing international students’ perceived deficiencies, particularly language barriers (Y. Y. Jin et al., 2021), while paying less attention to their capacity for proactive problem solving. For example, support programs for international students have often emphasized academic writing skills to facilitate academic adjustment (M. Kang, 2020; Y. Lee, 2020), general education courses such as Korean culture to support cultural adjustment (Mun, 2020; J. Kim et al., 2020), or the establishment of separate departments for international students to reduce academic gaps between local and international students (J. Y. Shin et al., 2018).

This deficit-oriented perspective tends to foreground students’ limitations and position external support as the primary means of addressing them. Such a tendency may be related to the broader policy context of international student recruitment in South Korea. The Study Korea Project series has been criticized for prioritizing the expansion of international student recruitment while giving insufficient attention to the quality of students’ academic experiences and support systems (Y. Kim, 2024). For instance, although TOPIK level 4 is commonly used as the minimum language proficiency requirement for admission, the average fulfillment rate across Korean universities has been reported to be only about 42% (Korean University Information System, 2025a). This suggests that the rapid expansion of international student recruitment in Korean higher education has, in many cases, outpaced applicants’ actual readiness in terms of language proficiency and academic competencies. This gap between admission requirements and actual proficiency levels translates, at the individual level, into students struggling with basic course comprehension—an experience that in turn reinforces a deficit-oriented narrative in which educational institutions frame their role primarily as compensating for students’ linguistic and cultural shortcomings.

Yet paradoxically, our findings indicate that it is precisely this adversity that activates students’ agency: Faced with these structural gaps, international students often mobilize both internal resources, such as prior learning strategies, motivation, and resilience, and external resources, such as peer networks and institutional support, to cope with and navigate a challenging academic environment. Taken together, these dynamics suggest that international students’ academic adjustment cannot be fully understood through a deficit lens alone but must also account for the agentic strategies students develop in response to structural shortcomings. This points to the need to move beyond a provider-centered approach that asks what institutions should offer, toward a more student-centered approach that considers how students’ agency, willingness, and capacities can be supported.

Practically, these findings suggest that Korean higher education institutions should avoid concentrating their resources solely on language and cultural adaptation programs. Equal attention should be given to initiatives that recognize and support student agency, such as student-led mentoring, psychological and career counseling, and academic skills workshops.

Theoretically, these findings call for a reconsideration of existing adjustment models. Previous research has often focused on how individuals respond to the demands of a new environment (Inouye et al., 2023), while giving less attention to how students actively draw on available resources and negotiate the conditions that shape their adjustment. Our findings suggest that academic adjustment is not a one-way process, but a dynamic and agentic one in which students mobilize both personal and external resources to navigate structural constraints.

## 4. Limitations and Conclusions

### 4.1. Limitations

This study has several limitations. First, although the participants came from five countries, including China and Vietnam, which are among the largest source countries of international students in South Korea, the sample did not include students from Nepal, Uzbekistan, or Mongolia, which also account for a substantial proportion of the international student population. In addition, the study may overrepresent students with relatively high Korean language proficiency. Nine of the sixteen participants had advanced Korean proficiency, whereas the average fulfillment rate of Korean language admission requirements across Korean universities has been reported to be only about 42% (Korean University Information System, 2025a). Furthermore, participants were recruited from six institutions, three of which were in Seoul. Therefore, caution is warranted when transferring these findings to the broader population of undergraduate international students in South Korea. Future research should include participants from more diverse national backgrounds and with varying levels of Korean language proficiency. Second, while data triangulation can enhance the credibility of qualitative findings, this study primarily relied on interview data. Future research could incorporate multiple data sources, such as observations, institutional documents and the perspectives of faculty members, to provide a more comprehensive understanding of the experiences of international students. Finally, although including participants from diverse backgrounds broadened the range of experiences represented, this study used an embedded single-case design. In this design, international students’ academic adjustment within South Korean higher education constituted the bounded case, and individual participants served as embedded units of analysis. The analysis focused on shared patterns of meaning across participants and did not systematically examine differences in terms of nationality, institution, gender or field of study. The small and uneven number of participants within each subgroup also limited the possibility of meaningful comparisons. Future research could conduct comparative analyses across embedded units or employ a multiple-case design to explore how academic adjustment differs according to participant and institutional background.

### 4.2. Conclusions

Nevertheless, this study builds on previous research by demonstrating how international undergraduates exercise agency within the institutional and sociocultural context of South Korean higher education. The findings provide empirical support for the multidimensional nature of academic adjustment. Participants did not experience this simply as language development or academic achievement; rather, they saw it as an ongoing process shaped by motivation, interpersonal relationships, institutional conditions, and self-regulated coping. By highlighting how students actively responded to academic, social and institutional challenges, the study moves beyond deficit-based accounts of international students. The findings suggest that support programs should not only address students’ perceived shortcomings but also strengthen their agency and capacity to navigate challenges.

## Figures and Tables

**Table 1:** Participant Information.

Case	Nationality	Gender	Age	Grade	Major	Korean	Length of Stay
1	Vietnam	Male	25	4	Political Science and International Relations	6	6
2	Vietnam	Female	22	2	Media and Communication Studies	4	3
3	Vietnam	Female	23	4	Korean Language and Literature	6	2.5
4	Vietnam	Female	22	2	Media and Communication Studies	5	3.3
5	Vietnam	Female	25	4	Korean Language and Literature	5	4
6	Indonesia	Female	22	3	Software Convergence	4	2.
7	USA	Male	26	3	Media and Communication Studies	1	4
8	Indonesia	Male	21	2	Interior Architecture and Design	3	3.5
9	China	Female	21	2	Economics	3	1
10	China	Male	22	4	Theatre and Film	6	4
11	China	Female	23	4	Business Administration	6	3
12	China	Male	22	4	Business Administration	6	4
13	China	Male	22	3	Business Administration	6	4
14	China	Male	22	4	Foodservice Management	4	4
15	China	Female	23	4	Korean Dance	3	5
16	Malaysia	Female	23	4	Applied Music	6	5

*Note.* Korean = Korean Proficiency (TOPIK); Length of Stay = Length of Stay in South Korea (Year); USA = the United States; TOPIK (Test of Proficiency in Korean) administered by South Korea’s National Institute for International Education (NIIED) for non-native Korean speakers. It is divided into six levels (1–6), with higher levels indicating greater proficiency.

**Table 2:** Interview questions.

No.	Questions
1	What does a “successful study-abroad experience” mean to you?
2	If you freely imagine yourself adjusting well academically, what would be the most important characteristics of your school life—your key indicators or criteria for judging that you are doing well?
3	How would you describe your current level of academic adjustment?
4	As a university student, you are expected to complete various academic tasks—such as attending classes, writing assignments, participating in class discussions, and giving presentations. What was your experience like as you carried out these academic tasks?
5	As a member of the university community, you interact with various people on campus. Please talk freely about your experiences communicating with different campus members—for example, professors, Korean students, students from your home country, and administrative staff.
6	When you face difficulties in your studies, what kinds of attempts or efforts did you make to overcome them?
7	Among the efforts you have made so far, what has helped with solving the problem, and what has not helped?
8	Have you ever used—or are you aware of—any support programs provided by your university for international students (e.g., academic/learning support, daily life support, career/employment support)? If you have not encountered them or don’t know about them, what do you think is the reason?
9	(If you have used them or know about them) Among the various support programs designed to help international students’ academic adjustment at Korean universities, which were helpful and which were not? Please explain why.
10	If you could start your study-abroad life again, what areas would you put more effort into in order to improve your academic adjustment?
11	What kinds of environmental or institutional support systems would you like to see in place to better support international students’ academic adjustment?

## Data Availability

The data are not publicly available due to ethical restrictions related to participant confidentiality. As the interview data contain identifiable information that could compromise participants’ anonymity, and as participants consented to the use of their data only for the purposes of this study, the raw data cannot be shared publicly. This restriction is consistent with the conditions approved by the Sejong University Institutional Review Board.

## Appendix A. Themes, Categories, and Concepts

**Themes**	**Categories**	**Subcategories**	**Concepts**
Becoming a Competent, Connected and Self-directed Student	Academic competency	Comprehension of course content	Reverse-coded: difficulty understanding instructors’ lectures. Familiarization with instructional/learning styles. Monitoring and managing key academic announcements and notices.
		Proficiency in the Korean language	Korean language fluency. Reverse-coded: limited Korean communicative competence.
		Objective academic attainment	Gradual grade improvement. Academic performance exceeding that of other international students. High academic achievement/excellent grades. Acquisition of new knowledge.
	Learning attitude and motivation	Diligence in studying	Conscientious engagement in coursework. Timely problem solving (non-procrastination). Intentional verbal participation in small-group activities. Time investment for assignments and examinations. Additional time devoted to difficult content. Preview/review practices to enhance class comprehension.
		Enjoyment of learning	Class engagement driven by interest rather than grade preoccupation. Enjoyment of the learning process. Making a deliberate effort to approach studying with a sense of ease and composure.
		Maintenance of self-efficacy	Perceived ability to complete assignments independently. Reverse-coded: negative affect when studying is difficult (irritation/anxiety/self-dissatisfaction). Confident expression of one’s ideas in class. Reverse-coded: low learning motivation and tendency to give up easily. Reverse-coded: difficulty collaborating/discussing with Korean peers in group work. Comfortable participation and task fulfillment in team projects.
	Social interaction and relationships	Building positive relationships with faculty and peers	Natural socialization with Korean students. Friendship formation with international students from diverse countries. Proactive communication with peers. Maintaining a good relationship with professors to feel more comfortable in academics.
		Autonomy-oriented social adjustment	Pragmatic compromise regarding “full integration.” Reverse-coded: overdependence on peers as maladaptive adjustment. Avoidance of inefficient socializing.
	Self-acceptance and self-directed growth	-	Self-acceptance and personally meaningful goal setting Openness to Challenges and Positive Mindset Adjustment Personal Growth and Self-improvement
	Environmental adaptation	Adaptation to the academic environment	Adjustment to Korean University Learning Culture Participation in diverse on-campus activities. Reverse-coded: insufficient efforts to adapt to new environments.
		Adaptation to the living environment	Balancing part-time work and academic demands. On-campus teaching assistant experience.
Negotiating Adjustment amid Barriers and Support	Risk factors	Individual factors	Reduced academic self-efficacy. Uncertainty about major/field. Health-related challenges.
		Social factors	Acculturative stress. Difficulty socializing with Korean students. Emotional isolation. Influence of academically passive compatriot peers.
		Academic factors	Limited professor support. Language barriers limiting class participation. Difficulty adapting to Korean educational systems.
		Financial factors	Difficulties in scholarship application. Financial strain undermining academic focus.
	Protective factors	Support from Korean student peers	Social affiliation with Korean peers. Norms of mutual respect. Inclusive attitudes toward international students.
		Support from professors	Professors’ interest/attention. Professors’ kindness and supportive attitudes.
		Financial support	Relatively easy access to part-time work. Scholarship access/application.
		Systematic/Institutional support	National health insurance coverage. Campus facilities and instructional environments. International-student-only courses. International-student-friendly systems. Institutional support programs (including student life, academic, career, and mental health services, etc.)
Taking Ownership through Action and Reflection	Active coping	Sustaining academic diligence	Preview and review routines. Consistent studying habits. Establishing a stable daily routine. Thorough preparation for presentations. Greater time/energy investment relative to Korean peers. Maintaining concentration during class.
		Enhancing Korean competence	Clarification-seeking from Korean peers for unfamiliar expressions. Korean learning via Korean media/entertainment. Efforts to learn/experience Korean culture. Ongoing discipline-specific Korean language learning.
		Self-regulated learning strategies	Exploration of personally effective learning methods. Goal setting and sustained engagement. Independent information seeking and utilization for comprehension. Self-directed problem solving. Time-management strategies Self-directed remediation of academic weaknesses.
		Proactive course registration	Strategic course registration. Changing language of instruction when necessary.
		Seeking active support	Help-seeking from professors. Proactive interaction with peers. Help-seeking from Korean friends/mentors. Help-seeking from senior international students. Private tutoring/external lessons.
	Reflective coping	Increased academic commitment	Research experience in a professor’s lab. Increased effort in English study. More proactive academic posture.
		More active social interaction	Initiating contact with peers and faculty. Maintaining close ties with senior compatriots.
		Pre-departure Korean preparation	Strengthening Korean competence prior to studying abroad.
		Pre-departure information seeking	Major exploration prior to decision-making. More systematic learning plans. More thorough investigation of campus life (e.g., club information).
*Note.* Items labeled “Reverse-coded” indicate negatively framed concepts			

## References

[B1-behavsci-16-01390] Ahn H. S., Kim W., Kim E. (2023). Effect of developing and implementing a learning support program for international students on their achievement and university life adjustment. The Korean Journal of Educational Methodology Studies.

[B2-behavsci-16-01390] An H., Jang Y. (2024). Helpful and unhelpful factors in psychological counselling as perceived by Chinese international students. The Korean Journal of Counselling and Psychotherapy.

[B3-behavsci-16-01390] Anderson P. H., Lawton L. (2015). The MSA: An instrument for measuring motivation to study abroad. Frontiers: The Interdisciplinary Journal of Study Abroad.

[B4-behavsci-16-01390] Bak J. H. (2024). A study on the successful study adaptation of foreign students in Korea: Based on a survey of international students at J university. Korea and World Review.

[B5-behavsci-16-01390] Baker R. W., Siryk B. (1984). Measuring adjustment to college. Journal of Counseling Psychology.

[B6-behavsci-16-01390] Braun V., Clarke V. (2006). Using thematic analysis in psychology. Qualitative Research in Psychology.

[B7-behavsci-16-01390] Chen X., Kang J. R. (2018). The effects of academic self-efficacy and academic emotion regulation on academic achievement among Chinese students in Korea: Mediating effect of achievement goal orientation. Journal of Learner-Centered Curriculum and Instruction.

[B8-behavsci-16-01390] Galanova D. (2018). A study on the academic experiences of Uzbek international students enrolled in Korean universities. The 13th Conference of Korean Language and Culture Education Society.

[B9-behavsci-16-01390] Gu Q., Schweisfurth M., Day C. (2010). Learning and growing in a ‘foreign’ context: Intercultural experiences of international students. Compare: A Journal of Comparative and International Education.

[B10-behavsci-16-01390] Ha C. H., Zhu H. Q. (2016). Factors affecting academic achievement of Chinese students- focused on the Korean factors and teaching-learning method factors. Journal of Korean Culture.

[B11-behavsci-16-01390] Hong H. J., Hyun S. H., Jung S. Y., Jung C. W. (2013). A case study on the development and application of learning strategies for adaptation to college life of international students’ education program. The Journal of General Education.

[B12-behavsci-16-01390] Hong S. (2019). A study on the systematization curriculum of Korean language for academic purpose. Korean Language & Literature.

[B13-behavsci-16-01390] Hwang D. G., So R. N., Park C. (2016). A narrative inquiry of academic adjustment of Chinese engineering students placed on academic probation. Journal of Narrative and Educational Research.

[B14-behavsci-16-01390] Inouye K., Lee S., Oldac Y. I. (2023). A systematic review of student agency in international higher education. Higher Education.

[B15-behavsci-16-01390] Jang K. O. (2018). Convergence research on academic adjustment of foreign students. Journal of the Korea Convergence Society.

[B16-behavsci-16-01390] Jeong Y. R. (2018). A study on the international students’ difficulties in university and daily lives. Contemporary Society and Multiculture.

[B17-behavsci-16-01390] Jin H., Chi E. (2017). Development and validation of the dropout intention scale for Chinese students studying in Korean universities. Journal of Educational Evaluation.

[B18-behavsci-16-01390] Jin X., Hahm S. W. (2018). The effect of professor’s transformational leadership on Chinese students’ satisfaction with university, professor, and academic in Korea. Asia-Pacific Journal of Multimedia Services Convergent with Art, Humanities, and Sociology.

[B19-behavsci-16-01390] Jin Y. Y., Xu N., Ahn H. S. (2021). International students and academic adjustment: Research trends in South Korea from 2011 to 2020. Multicultural Education Studies.

[B20-behavsci-16-01390] Kang M. (2020). Study on how to run and improve educational writing for foreign students. The Journal of Society for Humanities Studies in East Asia.

[B21-behavsci-16-01390] Kang Y. S., Lee H. J. (2016). A phenomenological study on the differences of the perceived personal and institutional multicultural competence between professors and international students in Korea. The Journal of Multicultural Society.

[B22-behavsci-16-01390] Kim D., Kim A. Y., Kang E. (2007). The inquiry of Chinese graduate students’ adaptive experience in Korea: By grounded theory approach. Asian Journal of Education.

[B23-behavsci-16-01390] Kim H. J., Cho S. H. (2018). Design and effect of learning coaching program for improving learning capabilities of foreign students and north Korean defector university students. Contemporary Society and Multiculture.

[B24-behavsci-16-01390] Kim J. (2013). Exploring the study experiences of southeast Asian students at a Korean university in Seoul. The Southeast Asian Review.

[B25-behavsci-16-01390] Kim J., Ryu S., Kang M. S., Lee A. (2020). Developing a general education curriculum for international students: Focusing on the contents and components of academic competence. Korean Journal of General Education.

[B26-behavsci-16-01390] Kim J., Sim J. (2018). A research of the construct development for evaluating team presentations performed by undergraduate international students in Korea: Focused on the presentation of contents. Journal of Korean Language Education.

[B27-behavsci-16-01390] Kim Y. (2024). A study on the Korean government’s foreign student attraction policy and Korean language education. The Society of Hansung Language and Literature.

[B28-behavsci-16-01390] Korean Educational Development Institute (2025). Current status of international students in Korean higher education institutions in 2025.

[B29-behavsci-16-01390] Korean Educational Statistics Service (2025). Basic statistics of education.

[B30-behavsci-16-01390] Korean University Information System (2025a). Status of international students.

[B31-behavsci-16-01390] Korean University Information System (2025b). Student dropout statistics.

[B32-behavsci-16-01390] Lazarus R. S. (1976). Patterns of adjustment.

[B33-behavsci-16-01390] Lee M. J., Kim D. M., Jin S. M. (2019). A qualitative study on the Chinese students’ experiences of overcoming the academic probations at the Korean universities. Journal of Education and Culture.

[B34-behavsci-16-01390] Lee S., Na Y. (2018). A study on international students’ academic adjustment to college: A preliminary study of course development. Korean Journal of General Education.

[B35-behavsci-16-01390] Lee S. Y., Kim J. Y., Park E. Y., Na M., Augusto S. S., Kang M. J., Baek J. S. (2022). Service design for foreign students’ adaptation to college life: Focusing on social interaction with Korean students. Design Convergence Study.

[B36-behavsci-16-01390] Lee Y. (2019). The effect of learners’ motivation on academic achievement: Focusing on international students learning Korean in Korea. Korean Language and Literature.

[B37-behavsci-16-01390] Lee Y. (2020). A study on the operational performance and development plan of writing clinics for foreign students. Korean Journal of General Education.

[B38-behavsci-16-01390] Lee Y., Xu N., Hong S., Lee E., Yang E. (2022). A qualitative meta-summary of college maladjustment factors among Chinese international students in South Korea. The Korean Journal of School Psychology.

[B39-behavsci-16-01390] Lee Y. K. (2014). A study about acculturation amongst foreign exchange student’s Korean culture and graduate school. Bilingual Research.

[B40-behavsci-16-01390] Li X. X., Ahn D. (2016). Influence of acculturative stress on psychological well-being and academic adjustment of Chinese international students in Korea. Korean Education Inquiry.

[B41-behavsci-16-01390] Lim S. R., Lee J. A. (2017). The effect of life stress, cultural adaptation stress and academic satisfaction in Chinese students studying in the republic of Korea: Target on beauty major college students. Korea Academy Industrial Cooperation Society, Journal of Korea Academia-Industrial Cooperation Society.

[B42-behavsci-16-01390] Ministry of Data and Statistics (2025). Total fertility rate.

[B43-behavsci-16-01390] Mori S. (2000). Addressing the mental health concerns of international students. Journal of Counseling and Development.

[B44-behavsci-16-01390] Mun J. H. (2018). Study on improving learning ability to liberal education for foreign students. Asia-Pacific Journal of Multimedia Services Convergent with Art, Humanities, and Sociology.

[B45-behavsci-16-01390] Mun J. H. (2020). Composition and realization of Korean culture liberal arts subjects for international students. Journal of the Korea Contents Association.

[B46-behavsci-16-01390] Noh E., Lee C. (2024). The needs assessment on career education of foreign students. The Journal of Employment and Career.

[B47-behavsci-16-01390] Noh J. (2020). Analysis on factors related to the academic achievement of foreign exchange students visiting for academic purposes. Journal of Learner-Centered Curriculum and Instruction.

[B48-behavsci-16-01390] Oh H. Y., Lee Y. H. (2018). Difficulties of international students recognized by professionals and psychological support plan. Journal of the Korean Society for Wellness.

[B49-behavsci-16-01390] Oh Y. J., Park M. A. (2018). The role of international education quality assurance system (IEQAS) and its implications. Global Studies Education.

[B50-behavsci-16-01390] Park E., Park J. (2017). Developing a diagnose tool(inventory) for foreign student’s academic life satisfaction. Journal of Ethnic Studies.

[B51-behavsci-16-01390] Park J. A. (2020). The impacts of parental career expectations, psychological separation from parents, career maturity, and social support on the dropout intention among international students in Korea. Master’s thesis.

[B52-behavsci-16-01390] Park S., Choi E. K. (2020). Designing a customized non-curricular program for international students: Focusing on D university’s program. Journal of Education & Culture.

[B53-behavsci-16-01390] Parsons R. L. (2010). The effects of an internationalized university experience on domestic students in the United States and Australia. Journal of Studies in International Education.

[B54-behavsci-16-01390] Patton M. Q. (1990). Qualitative evaluation and research methods.

[B55-behavsci-16-01390] Ponterotto J. G. (2005). Qualitative research in counseling psychology: A primer on research paradigms and philosophy of science. Journal of Counseling Psychology.

[B56-behavsci-16-01390] Poyrazli S., Grahame K. M. (2007). Barriers to adjustment: Needs of international students within a semi-urban campus community. Journal of Instructional Psychology.

[B57-behavsci-16-01390] Shin D. H., Kim D. S. (2019). Effects of homeland-oriented friendship on Chinese students’ academic achievement and emotional adaptation in Korea. Multicultural Education Studie.

[B58-behavsci-16-01390] Shin D. H., Kim S. (2020). How have international students in Korea been studied? Application of text mining and topic modelling to understand research trends. Korean Journal of Educational Research.

[B59-behavsci-16-01390] Shin I. C., Han J. E., Park H. (2018). A study of the dropout rate of international students and its factors in South Korea. The Journal of Multicultural Society.

[B60-behavsci-16-01390] Shin J. Y., Lee H. S., Lee Y. H. (2018). The analysis on the perception of university field for the necessity and expected effect of the department for foreign students in Korean university. Asia-Pacific Journal of Multimedia Services Convergent with Art, Humanities, and Sociology.

[B61-behavsci-16-01390] Sung H. J., Sung N. K., Hong N., Lee J. H. (2018). A phenomenological study on the academic probation experience of international undergraduate students. Journal of Learner-Centered Curriculum and Instruction.

[B62-behavsci-16-01390] Temple B., Young A. (2004). Qualitative research and translation dilemmas. Qualitative Research.

[B63-behavsci-16-01390] Vanchinkhuu N., Shin Y. (2023). Academic adjustment of international students studying in South Korea: The global Korea scholarship program perspective. Educational Linguistics.

[B64-behavsci-16-01390] Williams C. T., Johnson L. R. (2011). Why can’t we be friends?: Multicultural attitudes and friendships with international students. International Journal of Intercultural Relations.

[B65-behavsci-16-01390] Yan W., Cui Y. (2016). The relationship between Korean proficiency and Chinese students’ academic success in Korean universities. Teaching Korean as a Foreign Language.

[B66-behavsci-16-01390] Yin R. K. (2009). Case study research: Design and methods.

[B67-behavsci-16-01390] Yoon J. (2024). Fragmentary consideration on the extra-curricular program of employment support for international students. Studies on Humanities and Social Sciences.

[B68-behavsci-16-01390] Yoon J., Shim H. (2019). A study of reciprocal peer tutoring program for international students in university: Focused on actual operation cases. Teaching Korean as a Foreign Language.

